# Does the 10% Asymmetry Threshold Matter? Effects of Lower-Limb Asymmetries on Jumping and Agility in Basketball

**DOI:** 10.3390/jfmk10040445

**Published:** 2025-11-18

**Authors:** Nóra Szabó, Tamás Atlasz, Márk Váczi, Balázs Sebesi

**Affiliations:** 1 Doctoral School of Biology and Sport Biology, Faculty of Sciences, University of Pécs, Ifjúság Str. 6, H-7624 Pécs, Hungary; szabonori19@gmail.com; 2 Department of Sports Biology and Kinesiology, Institute of Sport Science and Physical Education, Faculty of Sciences, University of Pécs, Ifjúság Str. 6, H-7624 Pécs, Hungary; vaczi@gamma.ttk.pte.hu (M.V.); sebesi.balazs@pte.hu (B.S.)

**Keywords:** inter-limb asymmetry, basketball, countermovement jump, change of direction, agility, triple hop, performance testing

## Abstract

**Background**: Lower-limb asymmetry is linked to injury risk and may impair performance, yet evidence in basketball is inconsistent. A commonly cited 10% threshold is proposed as critical, but its practical relevance in basketball-specific tasks remains unclear. This study examined the effects of asymmetry on vertical jump and agility performance in basketball players, with particular focus on the 10% threshold. **Methods**: Male university basketball players (n = 20) completed unilateral jump tests (single-leg countermovement jump, single-hop, triple-hop, 6 m hop) and a bilateral COD (change of direction) test to quantify asymmetry. Basketball-specific performance was evaluated using the Lane Agility Test and bilateral countermovement jump. Asymmetry indices were calculated as absolute percentage differences. Paired tests, Welch’s *t*-tests (<10% vs. ≥10% asymmetry), and Pearson correlations were applied. **Results**: Significant inter-limb asymmetries were detected across all unilateral tasks (large effect sizes). Players with ≥10% asymmetry showed reduced bilateral countermovement jump height compared to <10% (*p* = 0.039, d = 1.00). Triple-hop asymmetry correlated strongly with slower Lane Agility Test times (r = 0.62, *p* = 0.003), while single-leg jump asymmetry correlated moderately and negatively with bilateral countermovement jump height (r = −0.46, *p* = 0.043). No significant associations were found for COD asymmetry. **Conclusions**: In the present study, inter-limb asymmetries exceeding 10% were associated with impaired vertical jump performance. Triple-hop asymmetry appears most relevant for agility, whereas COD asymmetry may not adequately reflect basketball-specific demands. Monitoring and reducing inter-limb asymmetries may support both performance and injury prevention.

## 1. Introduction

### 1.1. Physical Demands of Basketball

Basketball is a high-intensity intermittent team sport that requires players to perform frequent explosive actions such as sprinting, jumping, shuffling, and rapid changes of direction (COD), interspersed with periods of lower-intensity activity [1,2,3]. Time–motion analyses indicate that basketball players perform 40–50 explosive vertical jumps per game, including actions such as jump shots, layups, rebounds, and blocks [4,5,6,7]. In addition, players execute high-intensity COD approximately every 2.56 s of live play [8], with frequent accelerations exceeding 4 G (33.6/min), 6 G (9.1/min), and even 8 G (2.3/min) during decelerations, landings, and defensive contacts [9]. The ability to repeatedly perform these sport-specific tasks is underpinned by neuromuscular qualities such as explosive strength, reactive power, agility, and coordination, which are considered critical determinants of basketball performance [10,11,12].

### 1.2. Concept and Mechanisms of Inter-Limb Asymmetry

A growing body of evidence highlights inter-limb asymmetry as a factor that may compromise these neuromuscular demands. Most athletes exhibit a dominant limb, typically used preferentially for actions such as jumping or kicking, which can lead to systematic differences in force or power production between legs [13,14,15,16]. However, the identification of dominance is not always consistent: nearly half of athletes may perform better on the limb they did not self-report as dominant, raising concerns about the reliability of dominance-based classifications [17,18]. This inconsistency highlights the importance of using performance-based assessments rather than self-reported dominance, as applied in the present study, to ensure a more objective evaluation of inter-limb asymmetry. As a result, performance-based assessments are increasingly recommended to capture the true extent of asymmetry. Importantly, larger inter-limb discrepancies have been associated with increased injury risk and potential impairments in motor efficiency [19,20,21,22].

Inter-limb asymmetries may impair performance by disrupting neuromuscular coordination during bilateral tasks. When force or movement timing differs between limbs, compensatory strategies may emerge, leading to suboptimal motor unit recruitment and reduced overall efficiency of force production. Such alterations can compromise synchronization between the lower limbs, ultimately limiting performance in explosive, bilateral movements such as jumping or COD tasks [23,24,25].

### 1.3. Evidence Supporting the 10% Threshold

While inter-limb asymmetry has traditionally been examined in the context of injury prevention, there is growing recognition of its potential impact on athletic performance. Neuromuscular asymmetry can alter balance, joint stability, and movement coordination, which may directly affect key basketball skills such as COD and vertical jumping [26,27]. Several studies have therefore supported the use of a 10% inter-limb asymmetry threshold as a meaningful cut-off point for potential performance impairment. For instance, Bishop et al. [28] reported that asymmetries greater than 10% were associated with impaired jump performance and slower COD speed. Similarly, Bell et al. observed that exceeding this threshold had a negative influence on agility-based tasks [29]. Such findings suggest that asymmetry may have dual implications, both increasing susceptibility to injury and impairing sport-specific performance.

### 1.4. Evidence Challenging the 10% Threshold

However, other evidence challenges the universal applicability of this threshold. Some studies indicate that elite athletes often present with higher levels of asymmetry than non-elite athletes, likely as a consequence of long-term sport-specific specialization, yet without consistently detrimental effects on performance [30,31]. In contrast, other research in sports that emphasize strength and balance, such as football, tennis, handball and cycling, has reported clear performance impairments associated with asymmetry [32,33,34,35,36,37]. These examples are included for contextual comparison only, as the movement symmetry demands and kinetic patterns of these sports differ from those in basketball. Among athletes participating in these sports, inter-limb asymmetry has been observed within a range of about 8% to 15%. These conflicting results indicate that the 10% threshold may not represent a universal cut-off, and its impact could be influenced by sport-specific movement patterns, testing protocols, and the nature of the task being assessed.

### 1.5. Specific Findings in Basketball

In basketball, however, the evidence is less consistent, and the direct influence of lower-limb strength asymmetry on fundamental performance indicators such as COD speed and vertical jump height remains underexplored. Some studies suggest that the commonly cited threshold of 10% asymmetry may not necessarily impair performance in young basketball athletes, as sprint, agility, and jump outcomes were unaffected despite exceeding this value [38]. Similarly, investigations examining asymmetries in jump performance across different planes of motion found no significant associations with multidirectional speed [35]. Moreover, strength imbalances measured at the knee and hip joints using isokinetic dynamometry were not significantly related to vertical jump performance [39]. Collectively, these findings illustrate the inconsistent relationship between inter-limb asymmetry and basketball-specific performance, underscoring the need for further targeted research in this sport.

### 1.6. Practical Relevance, Research Gap, and Study Aim

Given the high frequency and importance of explosive jumps and COD maneuvers in basketball, understanding the relationship between inter-limb asymmetry and these performance measures has significant practical implications. Clarifying this relationship could support coaches, strength and conditioning specialists, and sports medicine practitioners in designing targeted interventions to reduce asymmetry, thereby enhancing both performance and resilience to injury. Furthermore, the prevalence of unilateral actions and asymmetrical movement patterns inherent to basketball practice may predispose athletes to developing greater inter-limb asymmetries compared to participants in other sports [40,41]. For example, the Limb Symmetry Index has been reported as 94% in male soccer players and 95% in volleyball players, but only 81% in basketball players, indicating a substantially higher degree of asymmetry in basketball [41].

Although lower-limb asymmetry has been widely studied, its direct impact on basketball-specific performance remains unclear. Some findings suggest decrements in jump and COD performance beyond a 10% asymmetry threshold, while others report no significant associations. Given that basketball players often display greater asymmetry than athletes in other sports, it is particularly important to determine whether surpassing this commonly cited 10% cut-off indeed translates into measurable performance deficits. To address this gap, the present study directly compared athletes with asymmetry levels below and above the 10% threshold in order to clarify its practical relevance for basketball performance.

Therefore, the purpose of this study was to examine the impact of lower-limb strength asymmetry on basketball-specific performance outcomes, namely vertical jump height and COD speed. Based on prior evidence, we hypothesized that: (1) greater lower-limb asymmetry would negatively affect COD performance; (2) asymmetries exceeding 10% would be associated with greater decrements in COD speed; (3) greater inter-limb asymmetry would be associated with lower bilateral vertical jump performance across the full sample; and (4) subjects exhibiting inter-limb asymmetries exceeding 10% would demonstrate significantly greater reductions in vertical jump height compared to those below this threshold. Confirming these hypotheses would address an important gap in the literature and provide actionable insights for applied basketball training and rehabilitation practices. Specifically, the findings may assist coaches and practitioners in optimizing training load distribution, designing individualized strength and conditioning programs, and implementing targeted asymmetry monitoring protocols to enhance performance and reduce injury risk.

## 2. Methods

### 2.1. Participants

Male university basketball players (n = 20) volunteered to participate in the study. All participants engaged in structured basketball training twice per week (90 min per session), regularly competed in a regional championship, and additionally performed two to four weekly conditioning or strength sessions as part of their training program. Their descriptive characteristics are presented in Table 1. All participants were free from musculoskeletal injuries at the time of testing, had a minimum of five years of competitive basketball experience, and had not sustained any serious lower-limb injury within the previous 6 months. None of the participants had a history of ACL reconstruction or other major lower-limb surgery. Participants with any neurological, cardiovascular, or chronic disorders were excluded.

### 2.2. Sample Size Calculation

We performed an a priori sample size calculation using G*Power 3.1.9.7, targeting a medium effect size (partial η^2^ = 0.06; approximately Cohen’s d ≈ 0.5), α = 0.05, and statistical power (1–β) = 0.80. This analysis indicated that a minimum of 12 participants would be required for the planned correlation analyses and within-subject limb comparisons. For subgroup analyses comparing athletes with <10% versus >10% inter-limb asymmetry (n = 10 per group), the statistical power would be limited for detecting medium effects. However, the study was specifically designed to capture large and practically meaningful differences—defined as performance-relevant asymmetry magnitudes that have been shown to produce measurable decrements in jump performance. Previous research has reported large effect sizes (Cohen’s d > 0.8) for such relationships [29] and power deficits of approximately 10% between limbs in collegiate athletes [42]. Accordingly, the present sample size (n = 20) provides adequate sensitivity for detecting differences of this magnitude.

### 2.3. Testing Procedures

Before testing, all participants completed a standardized, sport-specific warm-up lasting approximately 15 min. The protocol followed a three-phase structure: (1) general activation (~5 min) involving light jogging, side shuffles, and backpedaling drills; (2) dynamic mobility and strength preparation (~6 min) including dynamic stretches (e.g., walking lunges, leg swings, high knees, arm circles) and bodyweight calisthenics (e.g., squats, push-ups); and (3) specific neuromuscular activation (~4 min) consisting of progressive bounding, single-leg hops, and submaximal countermovement jumps. This sequence was designed to optimize athletes’ readiness for high-intensity basketball movements while simultaneously minimizing the risk of injury.

All measurements were conducted under standardized conditions, on the same indoor basketball court, at the same time of day, and with participants wearing their usual basketball shoes to minimize variability.

#### 2.3.1. Testing Asymmetry

Lower-limb asymmetry was assessed using a battery of single-leg jump tests adapted from Noyes et al. [22]. All jumps were performed twice with each leg, and the best attempt was used for analysis. Jump performance was measured with an optical measurement system (OptoJump Next, Microgate, Bolzano, Italy), which has been shown to be a valid and reliable tool for assessing jump performance [43].

The following tests were included:

Single-Leg Forward Hop (Noyes1): Participants performed a maximal single-leg hop for distance with both the right and left legs. Arm swing was restricted by placing the hands on the hips to minimize upper-body contribution.

Triple Hop for Distance (Noyes3): Participants executed three consecutive maximal single-leg hops, performed bilaterally. Distance was recorded using the OptoJump system.

6 m Hop for Time (Noyes6): Participants covered a 6 m distance with consecutive single-leg hops, and completion time was recorded with a handheld stopwatch.

Additionally, single-leg vertical jump height was assessed using the OptoJump system, performed bilaterally with hands on hips.

COD Test: COD ability was evaluated using a 10 m COD test adapted from Fort-Vanmeerhaeghe et al. [18]. Importantly, in this study, the COD test was specifically applied to quantify inter-limb asymmetry. Therefore, participants completed the drill in two conditions: once planting and changing direction with the right leg, and once with the left leg. Two flat rubber markers were placed 5 m apart to ensure safe cutting maneuvers. In each condition, participants sprinted from one marker to the other, performed a plant and directional change with the designated limb, and returned to the starting point. The total running distance was 10 m (5 m out and 5 m return), with a 180° change of direction performed at the far marker. Timing started from a standing start position, with the lead foot placed just behind the first timing gate. Both right-leg and left-leg conditions were tested twice, and the fastest trial in each condition was retained for analysis. One minute of passive rest was allowed between trials.

#### 2.3.2. Sport-Specific Performance Tests

Lane Agility Test: All participants started with one foot placed behind the start line, with no rocking or pre-movement allowed. Hand timing began with the first movement from the set position. Participants ran forward to the baseline, side-shuffled to the right across the baseline, backpedaled up the lane to the foul line, and then side-shuffled left back toward the start line. The completion time was recorded using a handheld stopwatch. Each participant performed two trials, and the best time was used for analysis.

Bilateral Countermovement Jump (CMJ): Explosive lower-limb power was measured through bilateral vertical countermovement jumps assessed with the OptoJump system. Participants performed two maximal jumps with their hands on their hips, separated by one minute of passive rest. Jump height was determined from flight time.

#### 2.3.3. Testing Order

All participants completed the testing battery in a fixed order: (1) inter-limb asymmetry tests, (2) COD test, (3) Lane Agility Test, and (4) bilateral countermovement jump. This order was applied consistently across all participants to ensure standardization.

### 2.4. Statistical Analysis

All statistical analyses were performed using GraphPad Prism (version 9.5.1, GraphPad Software, San Diego, CA, USA). Shapiro–Wilk test was applied to assess normality for each variable. Normally distributed paired data were analyzed with paired-sample *t*-tests, while non-normally distributed paired data were assessed using the Wilcoxon signed-rank test. For comparisons between groups with lower (<10%) and higher (≥10%) asymmetry, independent-samples *t*-tests with Welch’s correction were used to account for unequal variances. Effect sizes were calculated as Cohen’s d (or Hedges’ g for unequal groups) for independent comparisons, and as Cohen’s dz for paired tests. For non-parametric analyses, effect size estimates were expressed using Spearman’s r. Ninety-five percent confidence intervals (95% CI) were reported where applicable.

Pearson’s product–moment correlations were conducted to examine associations between asymmetry indices (calculated from COD, single-leg, and Noyes tests) and basketball-specific performance measures (Lane Agility Test and countermovement jump). When assumptions of normality were not met, Spearman’s rank-order correlations were applied.

Statistical significance was set at *p* < 0.05. In line with the preregistered hypotheses, four primary tests were conducted: (1) the effect of asymmetry on COD performance, (2) group comparisons for COD performance (<10% vs. ≥10% asymmetry), (3) the effect of asymmetry on CMJ performance, and (4) group comparisons for CMJ performance (<10% vs. ≥10% asymmetry). Therefore, no correction for multiple comparisons was applied to these confirmatory analyses. All other comparisons and correlation analyses were considered exploratory and should be interpreted with caution.

Inter-limb asymmetry was calculated as the percentage difference between limbs for each test, with the stronger and weaker limbs identified individually based on actual performance values. This performance-based approach provides a more accurate representation of asymmetry compared to dominance-based classifications.

## 3. Results

### 3.1. Descriptive Statistics

Descriptive statistics for unilateral performance tests are presented in Table 2, and sport-specific performance measures and asymmetry indices are summarized in Table 3.

### 3.2. Inter-Limb Asymmetries in Unilateral Performance Tests

Paired-samples analyses confirmed significant inter-limb asymmetries across all tasks, with consistently large to very large effect sizes. The observed mean differences were 0.11 s for COD performance (t(19) = 6.55, *p* < 0.0001, dz = 1.47, η^2^ = 0.69), 2.1 cm for single-leg CMJ height (t(19) = 5.80, *p* < 0.0001, dz = 1.30, η^2^ = 0.64), and 0.14 m for Noyes3 (t(19) = 13.28, *p* < 0.0001, dz = 2.97, η^2^ = 0.90).

For non-normally distributed variables, Wilcoxon tests confirmed significant inter-limb asymmetries: Noyes1 (median difference = 6.6 cm), W = 210, *p* < 0.0001, rs = 0.94, and Noyes6 (median difference = 0.07 s), W = 210, *p* < 0.0001, rs = 0.80.

### 3.3. Group Comparisons by Asymmetry Threshold (<10% vs. ≥10%)

**Figure 1:**
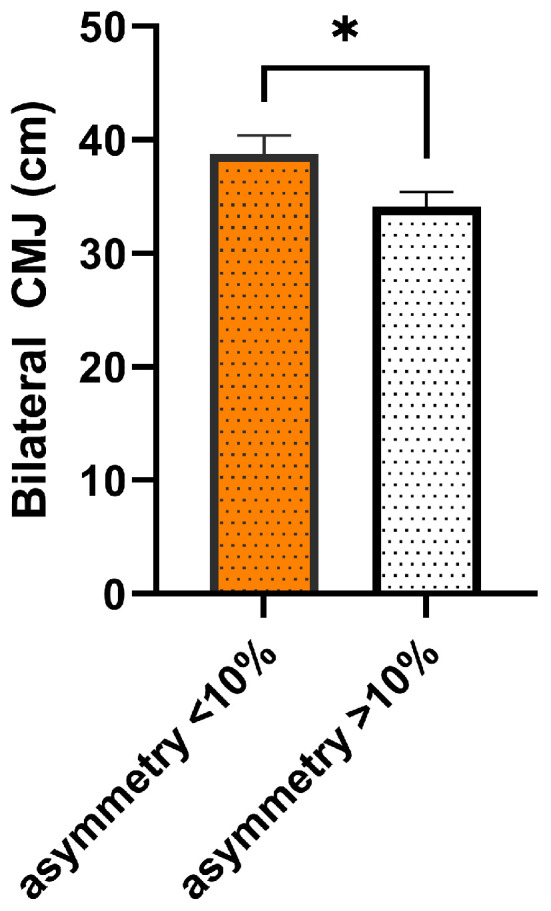
Comparison of bilateral CMJ height between <10% and ≥10% asymmetry groups. Bar graph comparing mean ± SEM bilateral countermovement jump (CMJ) height between athletes with low (<10%) (n = 10) and high (≥10%) (n = 10) inter-limb asymmetry. The asterisk indicates a significant between-group difference (*p* < 0.05).

Athletes with single-leg CMJ asymmetry >10% demonstrated significantly reduced bilateral CMJ height compared to those with <10% asymmetry (34.1 ± 3.9 vs. 38.8 ± 4.6 cm), Welch’s t(17.1) = 2.24, *p* = 0.039, Cohen’s d = 1.00 [95% CI: 0.07–1.93], Hedges’ g = 0.96, indicating a large effect (Figure 1).

### 3.4. Associations Between Asymmetry Indices and Basketball-Specific Performance

**Figure 2:**
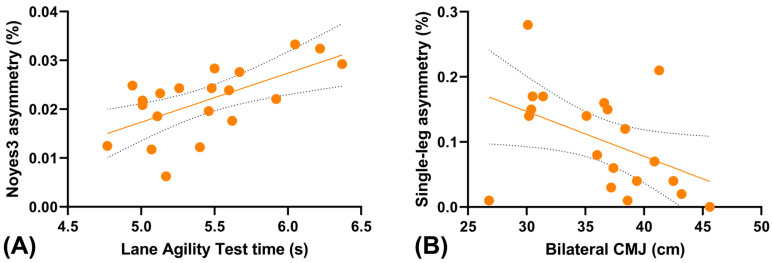
Relationships between inter-limb asymmetry and performance outcomes in basketball players. (**A**) Scatter plot illustrating the positive association between inter-limb asymmetry measured by the Noyes3 test and performance time in the Lane Agility Test (n = 20). Greater asymmetry was associated with slower agility-test performance. The solid line represents the line of best fit, and the dotted lines indicate the 95% confidence interval (CI). (**B**) Scatter plot showing the negative correlation between single-leg CMJ asymmetry and bilateral CMJ height. The regression line (solid) and dotted lines (95% CI) indicate that higher asymmetry corresponded to lower jump height.

Pearson correlations revealed that greater Noyes3 asymmetry was strongly associated with slower Lane Agility Test times (r = 0.62, 95% CI [0.25, 0.84], *p* = 0.003) (Figure 2). No significant associations were found between the Lane Agility Test and other asymmetry indices (r range = −0.46 to 0.23, all *p* > 0.05), with 95% confidence intervals within the range [–0.75, 0.61].

For bilateral CMJ performance, a moderate negative correlation was observed with single-leg CMJ asymmetry (r = −0.46, 95% CI [−0.75, −0.02], *p* = 0.043) (Figure 2), indicating that larger asymmetries were associated with reduced vertical jump performance. All other correlations were non-significant (r range = −0.47 to 0.23, all *p* > 0.05), and 95% confidence intervals for these associations are reported for completeness [–0.75, 0.61].

## 4. Discussion

The present study investigated the influence of lower-limb inter-limb asymmetry on basketball-specific performance outcomes, focusing on agility and vertical jump ability. The main findings were as follows. (1) Robust inter-limb asymmetries were consistently observed across all unilateral tests, with large to very large effect sizes; (2) athletes with asymmetries exceeding 10% demonstrated significantly reduced bilateral countermovement jump performance compared to those with <10% asymmetry; (3) greater Noyes3 asymmetry was strongly associated with impaired Lane Agility Test performance, while single-leg CMJ asymmetry showed a moderate negative correlation with bilateral countermovement jump height; and (4) contrary to expectations, COD-derived asymmetry did not relate to basketball-specific performance tests. Collectively, these findings provide new insights into the performance implications of inter-limb asymmetry in competitive basketball players.

### 4.1. Inter-Limb Asymmetry and Vertical Jump Performance

Consistent with prior work [28,29], our results demonstrate that inter-limb asymmetry exceeding the commonly cited 10% threshold can negatively influence performance, particularly bilateral countermovement jump height. This aligns with the notion that vertical jump ability depends critically on symmetrical force generation from both limbs, and imbalances may compromise take-off efficiency. This effect likely arises from disruptions in neuromuscular coordination between limbs, leading to less synchronized force production and reduced overall mechanical efficiency during jumping. When one limb contributes disproportionately, compensatory motor patterns may emerge that limit maximal jump height despite adequate unilateral capacity. From a practical standpoint, this emphasizes the relevance of unilateral jump training as both an assessment and a corrective tool for basketball players.

### 4.2. Inter-Limb Asymmetry and Agility

The significant association between Noyes3 asymmetry and slower Lane Agility Test times underscores that certain unilateral tasks are more sensitive indicators of basketball-specific agility. These findings extend earlier reports of negative associations between asymmetry and athletic performance in football, tennis, and handball [32,33,34,35,36,37], while contributing sport-specific evidence for basketball.

However, not all asymmetry indices demonstrated performance relevance. Specifically, COD asymmetry was not correlated with either the Lane Agility Test or the countermovement jump outcomes. This contrasts with earlier studies suggesting that COD performance may be impaired when asymmetry is high [28,29]. A likely explanation lies in the design of our COD test, which required two 5 m sprints with a single directional cut. As highlighted by Nimphius et al. [44], such tests are strongly influenced by linear sprinting ability and may therefore underestimate the true contribution of cutting mechanics. The concept of COD deficit—defined as the difference between COD test time and 10 m sprint time—has been proposed as a more isolated measure of cutting ability independent of sprint speed. Incorporating COD deficit in future basketball research may yield clearer insights into the relationship between inter-limb asymmetry and agility.

It is important to note that the first hypothesis was supported only for specific measures of asymmetry. Specifically, Noyes3 asymmetry showed a significant association with slower Lane Agility Test performance (r = 0.62), suggesting that unilateral jumping tasks may more effectively capture functionally relevant inter-limb imbalances that influence agility. In contrast, COD-derived asymmetry was not related to agility outcomes, likely due to the short 5 m test distance, which emphasizes linear acceleration rather than braking or cutting ability. This indicates that the relationship between asymmetry and agility performance is strongly dependent on the biomechanical demands of the test.

### 4.3. The Relevance of the 10% Threshold

Despite the modest sample size (n = 20), effect sizes were consistently large, strengthening the practical interpretation of our findings. Importantly, the magnitude of asymmetry observed in our cohort was comparable to previously reported values in basketball players, and unilateral vertical jump asymmetry exceeded the 10% threshold commonly cited as indicative of potential performance deficits [28,29]. It should also be noted that the magnitude of asymmetry differs across tests, as shown in Table 3, indicating that the 10% threshold may not represent an equally meaningful cut-off for all performance measures. Distance-based hop tests typically yield lower asymmetry values than power-based or velocity-dependent tasks, suggesting that test-specific thresholds may be warranted. Thus, the present sample can be considered representative of basketball athletes in this regard. From an applied perspective, the results suggest that monitoring and addressing asymmetry—particularly in unilateral jump tasks—may have meaningful implications for optimizing basketball performance.

These findings suggest that targeted strength and explosive power interventions could help reduce inter-limb asymmetry. Unilateral resistance training, plyometric drills emphasizing both stronger and weaker limbs (1:3 workload ratio), and eccentric overload exercises have been shown to effectively restore limb balance and enhance overall lower-limb power output [45,46,47,48]. Based on the present results, asymmetry in unilateral CMJ performance and the Noyes3 test appears to be the most sensitive indicator of basketball-specific performance capacity. Therefore, coaches and practitioners are encouraged to routinely monitor unilateral jump asymmetry as part of ongoing performance profiling and to implement corrective training strategies where discrepancies exceed approximately 10%.

### 4.4. Methodological Considerations and Future Directions

Given the cross-sectional design of this study, causal inferences cannot be drawn regarding whether reducing asymmetry directly enhances performance. To address this limitation, we recommend conducting longitudinal intervention studies in which athletes engage in targeted training programs aimed at reducing inter-limb asymmetry, accompanied by pre–post assessments of basketball-specific outcomes. Beyond agility and jumping, future investigations should also examine whether improvements in inter-limb balance translate into enhanced shooting accuracy (field-goal percentage), a skill that may be indirectly influenced by lower-limb symmetry through stability and force transfer during the shooting motion.

A limitation of the present study is the relatively small sample size used for the subgroup comparison (<10% vs. ≥10% asymmetry; n = 10 per group), which limits statistical power for detecting medium effects. Therefore, these results should be interpreted with caution and viewed primarily as indicative of large, practically meaningful differences rather than subtle effects. Additionally, no correction for multiple comparisons was applied to the four predefined hypothesis tests, as these were planned a priori and formed the confirmatory core of the study. All other analyses were considered exploratory and interpreted with caution, serving primarily to guide future research rather than to establish confirmatory evidence.

Furthermore, extending research to youth athletes is warranted. The adolescent period, particularly around peak height velocity, represents a sensitive developmental window in which neuromuscular imbalances may be more easily corrected. Early interventions could therefore mitigate long-term performance limitations and reduce the risk of injury. Finally, studies in female basketball players are crucial, as evidence suggests that women often display greater inter-limb asymmetries and may be more susceptible to associated performance decrements and injury risks. In addition, future research should examine the test–retest reliability of asymmetry measures, potential in-season fluctuations, and the identification of basketball-specific assessment protocols to enhance methodological consistency and practical applicability.

## 5. Conclusions

In summary, the present study demonstrates that inter-limb asymmetry is an important factor influencing basketball-specific performance, particularly bilateral vertical jump height and agility. Hypothesis 1 (greater asymmetry would negatively affect COD performance) and Hypothesis 2 (asymmetries ≥10% would further impair COD performance) were not supported. In contrast, Hypothesis 3 (greater asymmetry would negatively influence vertical jump performance) and Hypothesis 4 (asymmetries ≥10% would lead to greater impairments in bilateral jump performance) were supported. These results highlight that the impact of asymmetry depends on the specific test and movement task examined.

From a practical standpoint, we recommend regular monitoring of unilateral jump asymmetry—particularly in Noyes3 and single-leg CMJ tests—as part of ongoing performance profiling. When asymmetry exceeds approximately 10%, targeted interventions should be implemented, such as unilateral strength and plyometric training emphasizing the weaker limb to restore symmetry and optimize lower-limb power output. Overall, these findings reinforce the importance of individualized asymmetry management in basketball to enhance performance and resilience.

## Figures and Tables

**Table 1:** Descriptive characteristics of participants (n = 20).

Variable	Mean ± SD
Age (years)	23.9 ± 3.96
Height (cm)	187.7 ± 5.12
Body mass (kg)	91.0 ± 11.72
Training experience (years)	11.3 ± 5.05

**Table 2:** Descriptive statistics for unilateral performance tests (n = 20).

Test	Weaker Limb (Mean ± SD)	Stronger Limb (Mean ± SD)
COD time (s)	2.7 ± 0.17	2.6 ± 0.15
Single-leg CMJ (cm)	18.3 ± 3.90	20.4 ± 4.13
Noyes1 (cm)	121.7 ± 20.53	128.4 ± 19.89
Noyes3 (m)	6.5 ± 0.16	6.6 ± 0.18
Noyes6 (s)	2.4 ± 0.20	2.3 ± 0.16

Values are mean ± SD. COD = Change of Direction test; CMJ = countermovement jump; Noyes1 = single-leg hop for distance; Noyes3 = triple hop for distance; Noyes6 = 6 m timed hop. For each test, the stronger and weaker limbs were determined individually according to actual performance values, rather than predetermined laterality. Asymmetry indices were calculated as absolute percentage differences between limbs.

**Table 3:** Descriptive statistics for sport-specific performance tests and asymmetry indices (n = 20).

Variable	Mean ± SD
Lane Agility Test (s)	5.4 ± 0.44
Bilateral CMJ (cm)	36.4 ± 5.13
COD asym. (%)	4.0 ± 2.66
Single-leg asym. (%)	10.3 ± 7.75
Noyes1 asym. (%)	5.4 ± 3.00
Noyes3 asym. (%)	2.2 ± 0.71
Noyes6 asym. (%)	4.4 ± 3.41

Values are mean ± SD. COD = Change of Direction test; CMJ = countermovement jump; Noyes1 = single-leg hop for distance; Noyes3 = triple hop for distance; Noyes6 = 6 m timed hop. Asymmetry indices are expressed as percentages of inter-limb difference.

## Data Availability

The data presented in this study are available on request from the corresponding author due to privacy and ethical restrictions.
